# eGoT: enhanced graph-of-thoughts for multi-hop knowledge retrieval and hypothesis generation in biomedicine

**DOI:** 10.1093/bioinformatics/btag216

**Published:** 2026-07-07

**Authors:** Nihar Sanda, Benjamin M Gyori, Vito Quaranta, Auroop Ganguly, Ayan Paul

**Affiliations:** The Institute for Experiential AI, Northeastern University, Boston, MA 02115, United States; Khoury College of Computer Sciences, Northeastern University, Boston, MA 02115, United States; Department of Bioengineering, Northeastern University, Boston, MA 02115, United States; The Institute for Experiential AI, Northeastern University, Boston, MA 02115, United States; Bouvé College of Health Sciences, Northeastern University, Boston, MA 02115, United States; The Institute for Experiential AI, Northeastern University, Boston, MA 02115, United States; Department of Civil and Mechanical Engineering, Northeastern University, Boston, MA 02115, United States; The Institute for Experiential AI, Northeastern University, Boston, MA 02115, United States; Khoury College of Computer Sciences, Northeastern University, Boston, MA 02115, United States

## Abstract

**Motivation:**

Research on biological mechanisms and disease processes is limited by fragmented findings across unstructured text in publications. Question answering and hypothesis generation that can reason across multiple sources can overcome this limitation. However, Large language models (LLMs) are prone to inaccuracies and lack clear provenance to primary evidence. Retrieval augmented generation approaches that have provenance to the original source of evidence address these shortcomings. However, the response richness is dependent on the retrieval process design. Current approaches often fail to produce responses requiring multi-hop reasoning across multiple domains.

**Results:**

To address this, we propose eGoT, which combines automated knowledge graph construction from biomedical literature with a novel graph-of-thoughts approach to query the knowledge base and construct comprehensive responses to natural language questions. Given a corpus of documents, eGoT first uses an LLM-based pipeline to identify and normalize entities and relationships and constructs graph and vector databases. Given an input question, eGoT performs multi-round LLM-based querying of the databases to construct a response. Benchmarking on datasets like MultiHopRAG, HotpotQA, and Ultradomain demonstrates eGoT’s superiority over state-of-the-art retrieval methods, including HopRAG, SireRAG, HiRAG, and HippoRAG. We demonstrate eGoT on two biomedical use cases: (i) generate responses to domain expert-curated questions on small cell lung cancer using 1046 PubMed Central publications, and (ii) demonstrate eGoT’s ability to find plausible connections between Lupus and climate factors (UV exposure) that affect disease trajectory.

**Availability and implementation:**

https://github.com/NNeuralDynamics/eGOT.git.

## 1 Introduction

Understanding biological mechanisms and disease processes, and designing therapeutic interventions, remains a central challenge in biomedical research. A key barrier, recognized since the concept of “undiscovered public knowledge” (Swanson 1986), is that relevant findings are fragmented across a large body of publications (about 39 million cataloged in PubMed to date), with mechanistic insights, molecular data, and clinical observations scattered across disparate documents (Gopalakrishnan *et al.* 2019). Explaining complex phenomena or generating novel hypotheses often requires piecing together evidence from multiple sources and tracing multi-hop reasoning chains such as relationships between genetic variants to downstream phenotypic manifestations. Knowledge fragmentation is particularly acute for complex diseases where environmental, genetic, and physiological factors interact across traditional disciplinary boundaries. Computational methods that can reason over and synthesize knowledge from multiple sources have the potential to accelerate discovery by uncovering latent connections that would otherwise require extensive manual literature review. Decades of work in biomedical natural language processing have produced robust methods for extracting entities, relations, and events from scientific text (Islamaj *et al.* 2024, Wei *et al.* 2024). However, assembling these extracted annotations across multiple sources and performing multi-hop reasoning while preserving the contextual nuances from original studies remains a significant challenge (Bachman *et al.* 2023).

Large language models (LLMs) have emerged as powerful tools for natural language understanding and reasoning (Brown *et al.* 2020, Khashabi *et al.* 2020). However, LLMs face fundamental challenges in factual reliability (Bang *et al.* 2023, Yao *et al.* 2023a): their parametric knowledge is incomplete and subject to obsolescence, resulting in hallucinations (Bang *et al.* 2023, Yao *et al.* 2023a) when queried for domain-specific or up-to-date information.

Retrieval-augmented generation (RAG) relies on retrieval from sources external to model weights as a paradigm to address these limitations (Izacard *et al.* 2023, Ram *et al.* 2023). RAG grounds responses in primary evidence, thereby reducing hallucinations and improving verifiability (Lewis *et al.* 2020, Gao *et al.* 2023). Initial RAG methods used dense passage retrieval, which splits large documents into smaller chunks and performs similarity search over a query to retrieve relevant passages, with many subsequent improvements including query rewriting, query transformation, query expansion, and re-ranking. However, RAG systems that rely on dense vector-based retrieval, while effective for many open-domain QA tasks, struggle with (i) long-context reasoning, (ii) complex multi-hop inference, and (iii) integration of structured relationships between fragmented information (Chen *et al.* 2024, Zhang *et al.* 2024a). Hence, purely text-based RAG often produces shallow reasoning chains and fails to capture logical dependencies between entities (Weller *et al.* 2026). These limitations in capturing logical dependencies necessary for multi-hop reasoning have motivated the development of graph-based RAG approaches (Peng *et al.* 2026, Han *et al.* 2025, Zhang *et al.* 2025b), moving beyond unstructured text and semi-structured resources to structured graph representations.

Graph retrieval-augmented generation (graph-based RAG) overcomes these challenges by leveraging structured knowledge graphs (KGs) for retrieval. These KGs represent knowledge as entities and relations, naturally encoding semantic and logical dependencies that traditional vector-based RAG overlooks. By retaining relevant triples, subgraphs, or reasoning paths, graph-based RAG systems provide richer contextual grounding and support more complex reasoning (Edge *et al.* 2024, Peng *et al.* 2026, Han *et al.* 2025, Liang *et al.* 2025). This makes them particularly valuable for domains where entity relationships and structured knowledge are central. Nevertheless, graph-based RAG methods face significant limitations (Han *et al.* 2024, Peng *et al.* 2026, Zhang *et al.* 2025b). First, graph incompleteness hampers reasoning since extracted KGs are often sparse and miss crucial connections. Second, retrieval strategies are often too local or rigid: many graph-based RAGs expand neighborhoods heuristically, missing semantically important but structurally distant information. Third, integrating structured knowledge with unstructured text remains underdeveloped, limiting performance when queries require hybrid reasoning.

Early approaches such as GraphRAG (Edge *et al.* 2024) improve factual grounding but fall short on deep multi-hop reasoning, while LightRAG (Guo *et al.* 2024) and PathRAG (Chen *et al.* 2026) trade comprehensiveness for speed. Hierarchical systems like HiRAG (Zhang *et al.* 2024b) and hybrid methods such as ToG (Sun *et al.* 2024) and ToG2 (Ma *et al.* 2024) combine logical reasoning with textual grounding but still suffer from incoherence across heterogeneous knowledge sources. Additional work addresses evidence triple verification (Li *et al.* 2023), hallucination mitigation (Guan *et al.* 2024), and multi-hop traversal strategies (Liu *et al.* 2025). Overall, current graph-based RAG systems underperform on tasks requiring deep multi-hop reasoning and adaptive retrieval across large heterogeneous knowledge corpora (Liang *et al.* 2025, Luo *et al.* 2025).

Recent work has begun applying RAG and knowledge graph approaches specifically to biomedical question answering. KRAGEN (Matsumoto *et al.* 2024) combines knowledge graphs with graph-of-thoughts prompting for biomedical problem solving, while MedRAG (Xu *et al.* 2025) constructs hierarchical diagnostic knowledge graphs for clinical decision support, and i-MedRAG (Xiong *et al.* 2025) enables iterative follow-up queries to refine retrieval. However, these systems primarily target clinical diagnosis or require pre-existing curated knowledge bases rather than constructing graphs from primary literature; moreover, approaches like KRAGEN use single-pass query decomposition that limits reasoning to 1–2 hops, falling short of the deeper chains required to connect findings across molecular, clinical, and environmental domains.

Parallel to advances in graph-based retrieval, LLM reasoning has progressed from Chain-of-Thought prompting (Wei *et al.* 2022) through Tree of Thoughts (Yao *et al.* 2023b) to Graph of Thoughts (GoT) (Besta *et al.* 2024), which supports arbitrary reasoning dependencies including branching and aggregation operations. This graph-based reasoning structure naturally aligns with graph-structured knowledge representations and inspired our approach.

In this work, we propose *eGoT: enhanced graph-of-thoughts for knowledge graph-based RAG*, a novel algorithm that addresses these limitations by integrating iterative reasoning with graph-based retrieval. Unlike single-pass graph traversal methods, eGoT uses a graph-of-thoughts framework where an LLM iteratively generates, evaluates, and expands reasoning paths over the KG. By adopting and enhancing the GoT framework’s iterative thought exploration and multi-path reasoning capabilities to operate over KGs, eGoT leverages the complementary strengths of structured reasoning patterns and graph-based knowledge retrieval, enabling more comprehensive exploration of complex, multi-hop queries than traditional single-pass retrieval methods. Our method improves retrieval by leveraging the power of LLMs to split a given query into multiple thoughts, leading to exploration of concepts that are semantically correlated but not specified in the query. In particular, eGoT extends beyond prior biomedical approaches like KRAGEN by enabling iterative multi-hop exploration over knowledge graphs constructed directly from unstructured literature, rather than requiring pre-existing curated knowledge bases.

**The primary contributions of this work are** 

- The eGoT algorithm that enables adaptive exploration of relevant subgraphs, discovery of latent connections, and synthesis of multi-hop evidence across diverse contexts.
- Evaluation and comparison of responses that are not only more comprehensive but also more faithful to the knowledge sources due to a unification of structured graph knowledge with the reasoning capabilities of LLMs.
- Demonstration of eGoT on biomedical use cases: question answering on small cell lung cancer literature with expert-curated questions, and cross-domain hypothesis generation linking lupus pathophysiology with UV radiation and climate factors.

We benchmark eGoT on standard datasets (Ultradomain, HotpotQA, MultiHopRAG), exceeding state-of-the-art performance on key metrics. We then demonstrate eGoT’s capabilities on two biomedical applications: (i) a knowledge graph of 1046 small cell lung cancer publications from PubMed Central, where eGoT answers domain expert-curated questions about disease mechanisms with verifiable source attribution; and (ii) a cross-domain case study integrating lupus pathophysiology with climate science literature to discover mechanistic pathways linking UV radiation to autoimmune disease exacerbation. These applications illustrate eGoT’s ability to synthesize evidence across disciplinary boundaries, discover latent connections requiring multiple reasoning hops, and generate testable hypotheses grounded in primary literature.

## 2 Materials and methods

**Figure 1:**
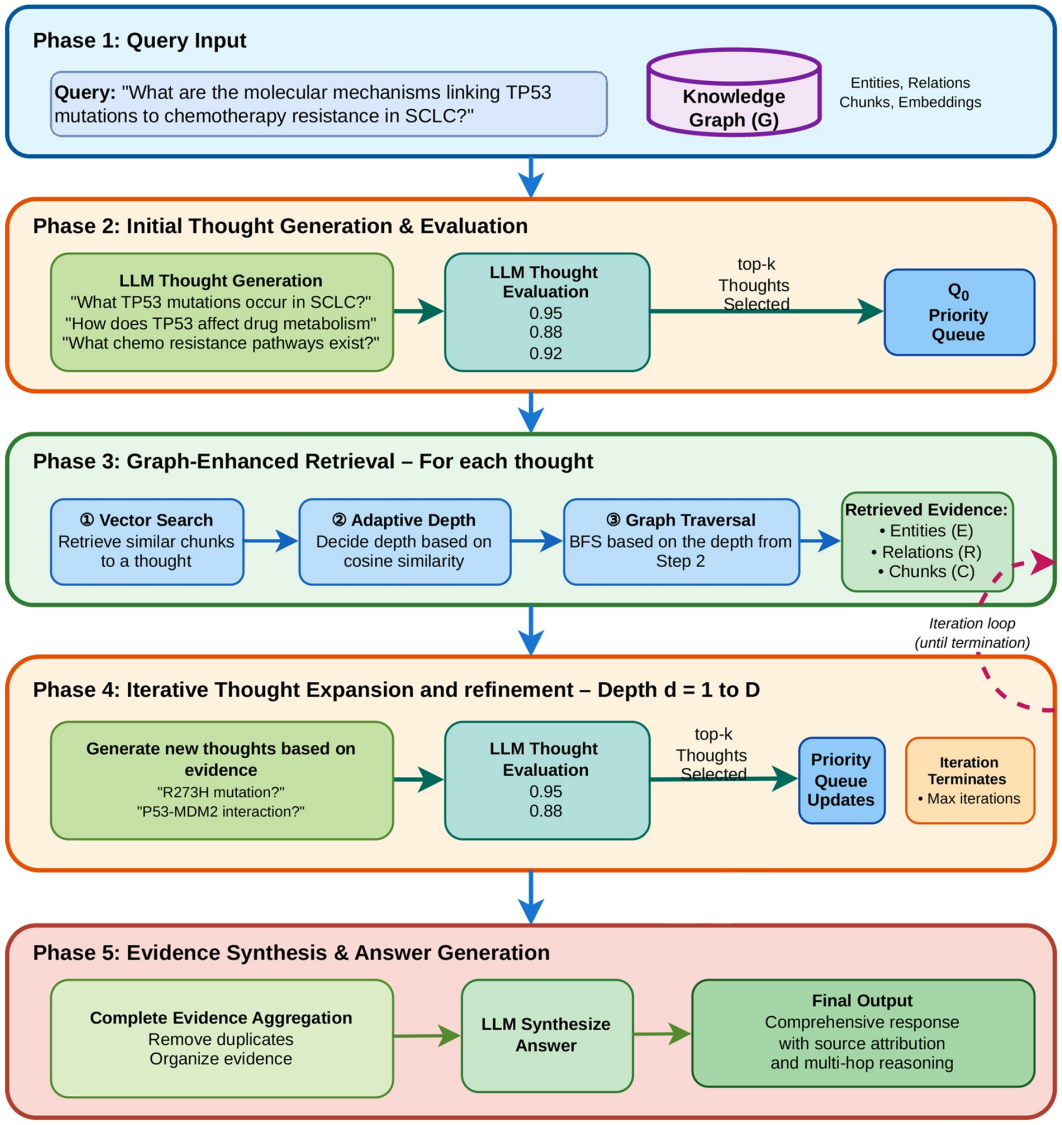
A schematic for eGoT retrieval algorithm.

Unlike traditional single-query GoT retrieval approaches, eGoT explores multiple reasoning paths in a graph, synthesizing evidence from multiple queries to construct answers. We design eGoT to be an enhanced GoT retrieval over graph-based RAGs, for graph-based retrieval systems that incorporate iterative thought exploration and multi-path reasoning. This approach uses graphs in two distinct roles: a GoT for performing structured reasoning during retrieval and a KG as a knowledge base for retrieving context from. The end-to-end retrieval and reasoning workflow of eGoT is summarized in Fig. 1, which depicts the interaction between thought generation, graph-enhanced retrieval, and iterative evidence synthesis. In this section, we delineate both our KG construction method and eGoT implementation.

### 2.1 Knowledge graph construction

We construct a semantically rich KG from unstructured documents in three key stages: entity-relation extraction, entity standardization, and relationship inference.

*Entity-relation triple extraction*: Following Carta *et al.* (2023), we begin by extracting entity-relation triples from input documents. Let $D=\{d_{1},d_{2},\ldots ,d_{n}\}$ be a collection of documents. We partition each document $d_{i}$ into overlapping text chunks $C_{i}=\{c_{i1},c_{i2},\ldots ,c_{im}\}$ where adjacent chunks share a context window of size *w*. For each chunk $c_{ij}$, we use a call to an LLM $L_{\text{extract}}$ with a single prompt to jointly extract entities and relations:


(1) $$V_{ij}=L_{\text{extract}}^{\text{entity}}(c_{ij}),\;\;E_{ij}=L_{\text{extract}}^{\text{relation}}(c_{ij},V_{ij}),$$

where $V_{ij}$ represents the set of entities extracted from chunk $c_{ij}$, and $E_{ij}$ represents the relations between pairs of entities within that chunk. The initial KG is then constructed as:


(2) $$G_{0}=\underset{i,j}{\cup }\{(h,r,t)|h,t\in V_{ij},r\in E_{ij}\}$$

*Entity standardization:* Raw entity extraction often produces multiple surface forms for the same conceptual entity. For instance, in biomedical text, “Systemic Lupus Erythematosus,” “Lupus,” and “SLE” may all refer to the same disease. We implement an entity standardization function $\phi :V_{\text{raw}}\to V_{\text{standard}}$ that maps entity variants to canonical forms. The standardization process operates in three stages:

1. *Normalization:* For each entity $e\in V_{\text{raw}}$, we apply text normalization, $e_{\text{norm}}=\text{lowercase}(\text{strip}(e))\setminus S$, where S represents a set of domain-specific stopwords.
2. *Clustering:* For each entity, we construct a set of similar entities based on lexical similarity and semantic equivalence:
  (3) $$C_{e}=\{e^{\prime }\in V_{\text{raw}}|\text{sim}(e_{\text{norm}},e_{\text{norm}}^{\prime })>\theta_{\text{sim}}\}$$
  where sim(·,·) is a similarity function combining string matching and semantic similarity, and $\theta_{\text{sim}}$ is a similarity threshold, where $\theta_{\text{sim}}$ is 4 characters.
3. *Representative Selection:* For each set $C_{e}$, we select a canonical form $e_{\text{canonical}}=\arg\max_{e^{\prime }\in C_{e}}\text{freq}(e^{\prime })\cdot \text{length}(e^{\prime })$ This process yields a standardized entity set $V_{\text{standard}}$ and a mapping function that ensures consistent entity representation across the KG.

*Hidden relationship inference:* After standardization, we enrich the KG by inferring latent relationships between entities that may not be explicitly stated in the text. This process uses both rule-based and LLM-based approaches.

*Rule-based Inference:* We apply transitive inference rules to discover implicit relationships: $R_{\text{trans}}=\{(h,r_{\text{inferred}},t)|\exists m:(h,r_{1},m)\in G_{0}\wedge (m,r_{2},t)\in G_{0}\}$.

Additionally, we add relationships $R_{\text{lex}}$ based on lexical patterns between entities sharing common roots or morphological variations as a heuristic for reconstructing ontologically related concepts that are not made explicit in text (for instance, “capitalism” and “capitalist decay”).

*Community-based Inference:* We identify disconnected components in the graph and use an LLM with a prompt in to hypothesize cross-community relationships, cf. Supplementary Information (SI):


(4) $$R_{\text{comm}}=L_{\text{infer}}(V_{{\text{comm}}_{i}},V_{{\text{comm}}_{j}})$$

where $V_{{\text{comm}}_{i}}$ and $V_{{\text{comm}}_{j}}$ represent entities from different graph communities. The enhanced KG is then:


(5) $$G=G_{0}\cup R_{\text{trans}}\cup R_{\text{lex}}\cup R_{\text{comm}}$$

*Graph structure organization:* Finally, we organize the KG with a hierarchical structure. Each triple (h,r,t)∈G is linked to its source chunk node $c_{ij}$, and each chunk node is linked to its parent document node $d_{i}$: $E_{\text{struct}}=\{(c_{ij},\text{HAS}\_\text{ENTITY},h),(c_{\text{ij}},\text{PART}\_\text{OF},d_{i})\}$.

For each chunk node, we also compute and store its vector embedding, ${\vec{c}}_{ij}=M_{\text{embed}}(c_{ij})$ where $M_{\text{embed}}$ is an embedding model. This hierarchical structure with embedded chunks enables efficient semantic search while preserving document-level context.

### 2.2 Retrieval using eGoT

The novel algorithmic flow of eGoT is given in Algorithms 1 and 2. Given the constructed KG, G, and a query *q*, our goal is to retrieve and synthesize relevant information by exploring multiple reasoning paths. We formulate this as an iterative thought generation and retrieval process that constructs an evidence graph $K_{q}={\left\{(t_{i},e_{i},s_{i})\right\}}_{i=1}^{n}$, where $t_{i}$ represents a thought, $e_{i}$ represents retrieved evidence, $s_{i}$ represents the relevance score (cf. Algorithm 1), and *n* is the total number of thoughts evaluated. We delineate the major components of this algorithm in what follows.

Algorithm 1 Graph-of-thoughts retrieval (eGoT)

**Input:** Query *q*, Knowledge Graph G, Maximum Depth *D*

**Output:** Final answer ${\text{answer}}_{\text{final}}$

1: $K_{q}\leftarrow \emptyset$       //Store all retrieved information

2:

3://Generate and evaluate initial thoughts

4:$T_{0}\leftarrow L_{\text{gen}}(q,¨\text{Initialquestion}")$

5:$T_{\text{eval}}\leftarrow L_{\text{eval}}(T_{0},q)$

6: $Q_{0}\leftarrow \text{top}-k(T_{\text{eval}})$     //Select top k thoughts

7:

8: **for**d=1**to** *D*

9: $Q_{d}\leftarrow \emptyset$          //Queue for next depth

10: **for all**$t_{i}\in Q_{d-1}$

11://Retrieve and process information

12:${\text{info}}_{i}\leftarrow R(t_{i},G)$

13:$a_{i}\leftarrow L_{\text{summ}}(t_{i},{\text{info}}_{i})$

14:$K_{q}\leftarrow K_{q}\cup \{(t_{i},a_{i},{\text{info}}_{i})\}$

15:

16://Build evidence from all retrieved info so far

17:$E_{\text{current}}\leftarrow \text{Format}(K_{q})$

18:

19://Generate and evaluate new thoughts

20.:$T_{\text{new}}\leftarrow L_{\text{gen}}(q,E_{\text{current}})$

21:$T_{\text{eval}}\leftarrow L_{\text{eval}}(T_{\text{new}},q)$

22:$t_{\text{best}}\leftarrow \text{top}-k(T_{\text{eval}})$

23:$Q_{d}\leftarrow Q_{d}\cup \{t_{\text{best}}\}$

24: **end for** 

25: **end for** 

26:

27://Synthesize final answer

28:$E_{\text{summary}}\leftarrow \text{FormatSummary}(K_{q})$

29:${\text{answer}}_{\text{final}}\leftarrow L_{\text{synth}}(q,E_{\;\text{summary}})$

30: **return**${\text{answer}}_{\text{final}}$

*Graph-of-thoughts generation:* Our approach begins by decomposing the original query into multiple exploratory thoughts. Given a query *q* we generate a set of thoughts $T_{0}=\{t_{01},t_{02},\ldots ,t_{0k}\}=L_{\text{gen}}(q)$ using an LLM call $L_{\text{gen}}$. The prompt for the generation can be found in the SI. Each thought $t_{0i}$ represents a distinct reasoning direction or hypothesis that could contribute to answering the original query. We prompt $L_{\text{gen}}$ to generate diverse thoughts that explore various aspects of a complex question.

*Thought evaluation and prioritization:* Not all generated thoughts contribute equally to answering the query. We use a thought evaluation mechanism using an LLM call $L_{\text{eval}}$ with prompt J to score each thought’s potential contribution:


(6) $$\text{score}(t_{ji})=L_{\text{eval}}(t_{i},q)\in [0,1]$$

Thoughts are then prioritized based on their scores, with higher-scoring thoughts explored first. The evaluation also enables efficient exploration of only the most promising reasoning paths while maintaining computational efficiency.

*Graph-enhanced retrieval:* Algorithm 2 is called by Algorithm 1 at this stage. For each selected thought $t_{i}$, we perform graph-enhanced retrieval over G. Our retrieval function R returns relevant entities $e_{i}$, relationships $r_{i}$, and document chunks $c_{ij}$:


(7) $$R(t_{i},G)={\left\{(e_{i},r_{i},c_{ij})\right\}}_{i=1}^{m}$$

The retrieval process uses a multi-stage approach that leverages both semantic similarity and graph structure to identify relevant information. We first retrieve candidate chunks using vector similarity search, then expand the retrieval scope through entity-centric graph exploration. Algorithm 2 is composed of three major parts

*1. Adaptive graph exploration:* A key innovation in our retrieval mechanism is the adaptive depth exploration based on semantic similarity scores. Given a query embedding $\vec{q}$, we dynamically adjust the exploration depth in the KG based on the cosine similarity between the query and entity embeddings:


(8) $$\text{depth}(e)=\{\begin{array}{c} 0\;\text{if}\;\;\text{cos}(\vec{q},\vec{e})<\theta_{a}\\ 1\;\text{if}\;\theta_{a}\leq \text{cos}(\vec{q},\vec{e})\leq \theta_{b}\\ 2\;\text{if}\;\;\text{cos}(\vec{q},\vec{e})>\theta_{b} \end{array}$$

where $0<\theta_{a}<\theta_{b}<1$ are two threshold parameters determining the depth used. This adaptive strategy ensures that highly relevant entities trigger deeper exploration of their neighborhoods, while maintaining computational efficiency by limiting exploration for less relevant entities.

*2. Graph traversal:* eGoT aggregates information at multiple levels: chunk-level for fine-grained textual content, entity-level for structured knowledge, and document-level for source attribution.

Algorithm 2 Graph-enhanced retrieval

**Input:** Query embedding $\vec{q}$, Knowledge graph G, Similarity threshold τ

**Output:** Retrieved entities *E*, relationships *R*, chunks *C*

1: $C_{\text{init}}\leftarrow \text{VectorSearch}(\vec{q},G,\tau )$ //Retrieve initial chunks

2: D←∅        //Documents

3:

4: **for all**$c\in C_{\text{init}}$

5:d←GetDocument(c)

6: D[d]←D[d]∪{c}   //Group chunks by document

7: **end for** 

8:

9: E←∅, R←∅, P←∅  //Entities, relationships, paths

10: **for all**chunks∈D.values()

11://Collect entities from chunks

12:$E_{\text{chunk}}\leftarrow \underset{c\in \mathrm{chunks}}{\cup }\text{GetEntities}(c)$

13:

14: **for all**$e\in E_{\text{chunk}}$

15:$\text{sim}\leftarrow \;\text{cos}(\vec{q},\vec{e})$

16: depth←GetDepth(sim)      //Adaptive depth

17:

18: if depth>0

19:$P_{e}\leftarrow \text{GraphTraverse}(e,\text{depth},G)$

20: $P\leftarrow P\cup P_{e}$            //Collect paths

21: **end if** 

22: **end for** 

23: **end for** 

24:

25://De-duplicate across all paths

26:$E\leftarrow \underset{p\in P}{\cup }\text{Nodes}(p)$

27:$R\leftarrow \underset{p\in P}{\cup }\text{Relationships}(p)$

28:

29://Format retrieval results

30:$\text{results}\leftarrow \text{FormatResults}(C_{\text{init}},E,R,D)$

31: **return** results

The GraphTraverse function implements a bounded breadth-first search that excludes meta-nodes (chunk and document) to focus on domain-relevant entities. The traversal respects relationship types, excluding structural relationships (HAS_ENTITY, PART_OF) to prioritize semantic connections.

*3. Result Aggregation and Formatting:* Retrieved information undergoes a structured aggregation process to create a comprehensive context for answer generation. For each document *d*, we construct a textual representation that combines:

1. *Chunk content*: The raw text from retrieved chunks, preserving their semantic relevance scores
2. *Entity descriptions*: Structured information about entities, formatted as Type: ID (description)
3. *Relationship triples*: Graph connections formatted as Subject: ID RELATION Object: ID

This multi-faceted representation ensures that the subsequent language model has access to both unstructured textual content and structured graph knowledge. The aggregated information for each thought $t_{i}$ is then processed through a context-aware language model, $a_{i}=L_{\text{summ}}(t_{i},R(t_{i},G))$ This approach enables eGoT to leverage the complementary strengths of vector similarity search and graph structure, providing rich context that supports answer generation across diverse query types. At this stage, $a_{i}$ is returned by Algorithm 2 to Algorithm 1. *Iterative thought expansion:* eGoT uses an iterative expansion strategy that progressively deepens the exploration of the KG. Unlike single-pass retrieval, our approach generates new thoughts based on accumulated evidence, enabling dynamic reasoning that adapts to discovered information. After the initial thought generation, the system maintains an evidence state $E_{j}$ that captures all previously gathered information:


(9) $$E_{j}=\overset{j-1}{\underset{i=1}{\cup }}\{(t_{i},a_{i},R(t_{i},G))\}$$

This evidence state serves as the context for generating subsequent thoughts, $T_{j}=L_{\text{gen}}(q,E_{j})$ where $T_{j}$ represents the new thoughts generated at iteration *j*, informed by both the original query *q* and the accumulated evidence $E_{j}$. To ensure efficient exploration, we maintain two key data structures: (i) a priority queue *Q* containing unexplored thoughts ranked by their evaluation scores and (ii) a set *P* of processed thoughts to prevent redundant exploration. The iterative process follows the following steps:

1. Select the top-*k* thoughts from *Q* based on their scores.
2. For each selected thought, perform retrieval and generate evidence.
3. Update the evidence state with new findings.
4. Generate new thoughts based on the updated evidence state.
5. Add novel thoughts to *Q* while updating *P* with processed thoughts.

This iterative expansion continues until one of the following conditions is met:

- The maximum number of iterations *N* is reached.
- The priority queue *Q* becomes empty (no more promising thoughts).
- The thought generation process produces no novel thoughts.

The iterative nature of eGoT enables emergent reasoning patterns where later thoughts can build upon insights from earlier explorations, leading to a more comprehensive understanding of complex queries.

*Evidence synthesis:* After iterative exploration, eGoT synthesizes the collected evidence into a final answer. The complete evidence graph $K_{q}$ contains all thought-answer-retrieval tuples:


(10) $$K_{q}=\overset{N}{\underset{j=1}{\cup }}\underset{t_{i}\in T_{j}}{\cup }\{(t_{i},a_{i},R(t_{i},G))\}$$

We aggregate the retrieved information by removing duplicates and organizing evidence by type (entities, relationships, documents, and answers). The LLM $L_{\text{synth}}$ then processes this aggregated evidence: ${\text{answer}}_{\text{final}}=L_{\text{synth}}(q,\text{Aggregate}(K_{q}))$

The synthesis LLM call $L_{\text{synth}}$ is used to (i) integrate insights from multiple reasoning paths, (ii) resolve contradictions between different evidence sources, (iii) structure the answer to address all query aspects, and (iv) maintain source attribution for verifiability.

This produces a coherent final answer that leverages the multi-path exploration while presenting information in a unified, comprehensive response. The dependence of eGoT on the choice of LLMs has been discussed in Section A, available as supplementary data at *Bioinformatics* online.

### 2.3 eGoT retrieval procedures

*Thought generation parameters:* We generate 2–4 thoughts per iteration to balance exploration breadth with computational efficiency.

*Iteration control:* For all the benchmarking results, we set a maximum depth of 2 iterations with only 3 thoughts explored per iteration, though the algorithm can terminate early if no new promising thoughts are generated.

*Graph enhanced retrieval parameters:* We use $\theta_{a}=0.3$ and $\theta_{b}=0.9$ as the two thresholds determining depth.

*Evidence aggregation:* Retrieved entities and relationships are aggregated across all thoughts, with duplicate removal to ensure comprehensive coverage of the KG.

## 3 Results

### 3.1 Benchmarking with standardized datasets

*Baselines:* We benchmark eGoT on the Query-Focused Summarization (QFS) and MultiHop QA (MHQA) tasks. We compare our results on these tasks with the current state-of-the-art RAG methods. For both benchmarks, we perform a comparison against alternative methods based on the availability of published results.

*Datasets: For the QFS task*, following Huang *et al.* (2025), we used three datasets from the Ultradomain benchmark: Legal and Agriculture, and Mix (Qian *et al.* 2025). Ultradomain is particularly relevant as a benchmark as it evaluates RAG systems across diverse applications on long-context tasks and high-level queries in specialized domains. Following Guo *et al.* (2024), we generate 100 questions for the Legal and Agriculture domains and 10 questions for the Mix domain. The questions are generated by giving a short description of the dataset to the LLM. *For the MHQA task*, we benchmark our system on 2 widely used datasets: MultiHopRAG (Tang and Yang 2024) and HotpotQA (Yang *et al.* 2018). For each of the tasks, we randomly sample 1000 questions and their passages. Additional details are in Table 2.

**Table 1:** Comparative evaluation of eGoT against other RAG methods with baseline across Legal, Agriculture, and Mix domains.^a^

	**Legal**		**Agriculture**		**Mix**	
	HiRAG	eGoT	HiRAG	eGoT	HiRAG	eGoT
Comp.	18.5	**81.50**	38.00	**62.00**	37.60	**62.40**
Emp.	17.00	**83.00**	21.00	**79.00**	43.40	**56.60**
Div.	26.00	**74.00**	37.50	**62.50**	48.00	**52.00**
Overall	17.00	**83.00**	30.50	**69.50**	42.20	**57.80**
	LightRAG	eGoT	LightRAG	eGoT	LightRAG	eGoT
Comp.	25.00	**75.00**	33.00	**67.00**	23.64	**76.36**
Emp.	15.00	**85.00**	19.50	**80.50**	13.17	**86.83**
Div.	37.00	**63.00**	37.00	**63.00**	18.99	**81.01**
Overall	20.00	**80.00**	29.00	**71.00**	16.27	**83.73**
	NaiveRAG	eGoT	NaiveRAG	eGoT	NaiveRAG	eGoT
Comp.	38.5	**61.5**	30.5	**69.50**	14.23	**85.77**
Emp.	19.5	**80.5**	19.50	**80.50**	13.07	**86.93**
Div.	29.50	**70.50**	27.50	**72.50**	11.53	**88.47**
Overall	30.50	**69.50**	23.50	**76.50**	12.30	**87.70**
	GraphRAG	eGoT	GraphRAG	eGoT	GraphRAG	eGoT
Comp.	47.50	**52.50**	41.00	**59.00**	26.74	**73.26**
Emp.	17.50	**82.50**	22.00	**78.00**	16.27	**83.73**
Div.	**52.50**	47.50	45.00	**55.00**	23.64	**76.36**
Overall	34.50	**65.50**	36.00	**64.00**	21.31	**78.69**
	KAG	eGoT	KAG	eGoT	KAG	eGoT
Comp.	18.00	**82.00**	15.50	**84.50**	8.10	**91.90**
Emp.	16.00	**84.00**	15.00	**85.00**	7.33	**92.67**
Div.	17.00	**83.00**	17.50	**82.50**	7.72	**92.28**
Overall	15.50	**84.50**	15.50	**84.50**	7.72	**92.28**
	No-GoT	eGoT	No-GoT	eGoT	No-GoT	eGoT
Comp.	40.5	**59.5**	38.50	**61.50**	44.20	**55.80**
Emp.	36.5	**63.50**	25.00	**75.00**	27.15	**72.85**
Div.	39.50	**60.5**	**51.00**	49.00	37.60	**62.40**
Overall	37.00	**63.00**	34.00	**66.00**	35.65	**64.35**

a We report percentage win rates of eGoT versus each competing method on Comprehensiveness (Comp.), Empowerment (Emp.), Diversity (Div.), and Overall performance metrics. The graph construction phase was done using LLaMA-4 Scout, while answer retrieval and generation were conducted using DeepSeek-V3. The bolded values denote the best performing model.

**Table 2:** Comparative evaluation of eGoT against various RAG methods on the HotpotQA multi-hop question answering dataset.^a^

Method	EM	F1	Reference
NaiveRAG	57.3	71.0	Huang *et al.* (2025)
RAPTOR	58.00	73.08	Zhang *et al.* (2025a)
GraphRAG	31.70	42.74	Zhang *et al.* (2025a)
LightRAG	25.00	43.20	Huang *et al.* (2025)
HippoRAG	59.9	74.29	Zhang *et al.* (2025a)
HiRAG	37.00	52.29	Huang *et al.* (2025)
SiReRAG	61.70	76.48	Zhang *et al.* (2025a)
HopRAG	61.30	**78.34**	Liu *et al.* (2025)
**eGoT**	**63.00**	71.11	

a We report Exact Match (EM) and F1 scores with the graph construction done using LLaMA-4 Scout, while answer retrieval and generation were conducted using GPT-4o. The bolded values denote the best performing models

*Evaluation metrics:* For benchmarking on standardized datasets, we followed previous works (Edge *et al.* 2024, Guo *et al.* 2024, Gutiérrez *et al.* 2024, Huang *et al.* 2025) for the evaluation methods. *For the QFS task*, we used the win rate to compare different methods with our method. The win rate tells us the percentage of times a given method generates higher-quality answers than its counterpart. This decision is taken by the LLM. The LLM judges the answer based on the following attributes: (i) Comprehensiveness: how thoroughly does the answer address the question, covering all relevant aspects and details? (ii) Empowerment: How effectively does the answer provide actionable insights or solutions that empower the user to take meaningful steps? (iii) Diversity: how well does the answer incorporate a variety of perspectives, approaches, or solutions to the problem? (iv) Overall: how does the answer perform overall, considering comprehensiveness, empowerment, diversity, and any other relevant factors? For a fair comparison, we also alternate the order of the answers generated by each pair of methods in the prompts and calculate the overall win rates of each method. The LLM used for judging (GPT OSS 120B) is never the same as the LLM that is used for KG construction and knowledge retrieval (LLAMA-4-scout/GPT-4o/DeepSeek-V3) throughout this work. *For the MHQA Task*, we evaluated the performance of eGoT and other methods using two standard metrics: exact match (EM) and F1 scores. EM measures the percentage of predictions that exactly match the ground truth answer after normalization (lowercasing, removing punctuation, and standardizing whitespace), providing a strict binary assessment of answer correctness. F1 Score, computed at the token level, measures the degree of overlap between predicted and ground truth answers, thus offering a more nuanced evaluation that gives partial credit for partially correct responses.

*Quantitative results:* In Table 1, we show a comparison of eGoT with several other retrieval methods. Our method clearly outperforms HiRAG (Zhang *et al.* 2024b), LightRAG (Guo *et al.* 2024), NaiveRAG (Gao *et al.* 2023), GraphRAG (Edge *et al.* 2024), and KAG (Liang *et al.* 2025) in all three domains and all four metrics, with only two exceptions. This clearly shows the superiority of our method in QFS tasks. In Tables 2 and 3, we compare eGoT with other retrieval methods on HotpotQA and MultiHopRAG datasets, respectively. While eGoT clearly outperforms several state-of-the-art methods on all metrics on the MultiHopRAG dataset, on the HotpotQA dataset, it outperforms only on exact match.

**Table 3:** Comparative evaluation of eGoT against various retrieval-augmented methods on the MultiHopRAG dataset.^a^

Method	Precision	Recall	F1	Accuracy
Naive	47.50%	59.90%	50.10%	60.40%
GraphRAG	50.10%	61.80%	52.60%	65.30%
LightRAG	44.80%	52.70%	46.40%	52.60%
PathRAG	45.30%	52.30%	46.80%	52.50%
HyperGraphRAG	50.30%	61.90%	52.60%	62.10%
TH-RAG	71.10%	72.00%	71.20%	72.20%
**eGoT**	**75.50%**	**79.20%**	**76.30%**	**79.10%**

a We report Precision, Recall, F1, and Accuracy scores with the graph construction being performed using LLaMA-4 Scout, while answer retrieval and generation are conducted using GPT-4o. The bolded values denote the best performing models.

*Ablation study:* We perform two ablation studies to establish the improvements seen in the benchmarking results. Firstly, we compare performance on QFS tasks with and without eGoT. These results can be found in Table 1 (and Table 1, available as supplementary data at *Bioinformatics* online). We see significant improvement when using eGoT as opposed to when not using it (No-GoT). This establishes the improvement in retrieval when using eGoT. Secondly, we test the improvement in retrieval that eGoT provides in larger knowledge graphs with a larger number of depth values. In Table 4, we compare three metrics after depth one of the algorithm versus depth three. These results highlight the fact that for a KG substantially larger than those obtained for common benchmarks (cf. Table 2, available as supplementary data at *Bioinformatics* online), a higher maximum depth number results in improved performance across all metrics. We have performed an ablation study for $\theta_{a}$ and $\theta_{b}$ (c.f. Supplementary Information) and $\theta_{a}=0.3$ and $\theta_{b}=0.9$ maximized the metrics of evaluation, namely (i) answer relevancy, (ii) faithfulness, (iii) context precisions, and (iv) context relevance. Additionally, we tested the removal of $L_{\mathrm{eval}}$ we saw a degradation of these metrics, especially context precision, as the algorithm chooses two thoughts at random and, hence, the performance is poor.

### 3.2 Case study: Small cell lung cancer

*Datasets:* The KGs in the benchmark datasets used above are relatively small (cf. Tables 3 and 4, available as supplementary data at *Bioinformatics* online). To better demonstrate the capabilities of eGoT to expand its reasoning and perform multi-hop retrieval, we created a KG for cancer biology. The dataset consists of 1046 full-text publications on small cell lung cancer (SCLC). These open-access publications were obtained from PubMed Central and were used for the KG construction.

*Evaluation metrics:* To test retrieval, we curate 21 questions of varying complexity with the help of an SCLC domain expert. The answers are evaluated using an LLM-as-judge approach for the following metrics (cf. Supplementary Information). (i) *LLM-based context precision without reference* leverages an LLM to assess whether each retrieved chunk contributed to the response generation, computing mean precision@k scores to quantify retrieval relevance without requiring ground-truth references. (ii) *Faithfulness* measures the factual consistency between generated responses and retrieved contexts by computing the ratio of claims in the response that are directly supported by the retrieved context to the total number of claims made, ensuring responses are grounded in the retrieved context. (iii) *Context relevance* evaluates the pertinence of retrieved contexts to the query by prompting an LLM with dual templates to assign relevance scores (0 = irrelevant, 1 = partial, 2 = complete), normalized to [0,1] and averaged to quantify query-context alignment.

*Results:* Performance results are shown in Table 4.

### 3.3 Case study: cross-domain integration for lupus and UV

To demonstrate eGoT’s capability for interdisciplinary biomedical reasoning, we conducted a case study investigating connections between environmental factors (UV radiation) and autoimmune disease (Systemic Lupus Erythematosus, SLE). This exemplifies a common challenge in computational biology: integrating knowledge across traditionally siloed domains to generate testable hypotheses about complex disease mechanisms.

*Motivation:* Understanding environment-disease relationships requires synthesizing knowledge from disparate fields including climate science, photobiology, immunology, and clinical medicine. While UV exposure is a known lupus trigger with 70%–90% of patients reporting photosensitivity, the complete mechanistic pathway connecting atmospheric changes to immune dysregulation remains incompletely characterized. This represents an ideal test case for cross-domain knowledge graph retrieval, as it requires traversing multi-hop reasoning chains: *climate change* →*atmospheric composition* →*UV radiation* →*DNA damage* →*immune activation* →*autoimmune manifestations*.

*Datasets:* We constructed the knowledge graph integrating two domain-specific subgraphs:

- **Medical subgraph**: 100 papers on lupus pathophysiology, photosensitivity mechanisms, and immune responses
- **Environmental subgraph**: 20 papers on UV radiation patterns, climate modeling (CMIP6), and atmospheric chemistry

Graph integration preserved domain-specific relationships while establishing cross-domain connections through community-based relationship inference (Section 2.1). The resulting graph contained 31 028 entities and 598 220 relationships spanning immunology, photobiology, and climate science.

*Experimental design:* We evaluated four conditions:

**exp-1:** Core papers directly addressing lupus-UV interactions.
**exp-2:** Alternative papers on similar domains (robustness test).
**exp-3:** Out-of-context papers from unrelated fields (boundary recognition).
**exp-0:** LLaMA-4 Scout without retrieval (baseline parametric knowledge).

*Cross-domain connection discovery:* For the query *“How is UV related to Lupus?*,” eGoT successfully bridged environmental and immunological domains, retrieving the mechanistic chain: UV exposure → keratinocyte apoptosis → autoantigen exposure → immune complex formation → systemic inflammation. This multi-hop path spanned four reasoning steps across distinct subgraphs.

*Multi-hop hypothesis generation:* For *“Can UV forecasting affect SLE management?*,” eGoT synthesized evidence across climate models, photobiology, and clinical outcomes to propose: (i) UV forecasting could enable predictive risk models for flare prevention, (ii) climate-driven UV changes may alter disease prevalence patterns, and (iii) personalized UV exposure thresholds could inform precision medicine approaches. These hypotheses were grounded in retrieved literature rather than parametric knowledge.

*Knowledge boundary recognition:* When tested with unrelated papers (exp-3), eGoT correctly returned “no information available” responses, avoiding hallucinated connections. In contrast, the baseline LLM (exp-0) provided plausible but unverifiable generic responses.

*Quantitative comparison:* Table 5 shows eGoT’s advantage over the baseline across faithfulness (0.89 versus 0.52), context precision (0.71 versus 0.38), and source attribution (100% versus 0% of claims traceable).

**Table 4:** Performance evolution of eGoT across different maximum depth values on the small cell lung cancer dataset.^a^

Metric	Depth 1	Depth 3	**Total change 1** → **3**
Faithfulness	0.80	0.90	+0.10 (+12.4%)
LLM context precision.	0.52	0.66	+0.14 (+27.3%)
NV context relevance	0.76	0.92	+0.16 (+21.9%)

a We report faithfulness, LLM context precision, and non-vectorized context relevance metrics at maximum depth 1 and maximum depth 3 using LLaMA-4 Scout.

**Table 5:** Comparison of eGoT with knowledge graph retrieval (exp-1) versus baseline LLM without retrieval (exp-0) on the cross-domain Lupus-UV case study.^a^

Metric	eGoT (exp-1)	Baseline (exp-0)	Δ
Faithfulness	0.89	0.52	+0.37
Context precision	0.71	0.38	+0.33
Source attribution	100%	0%	+100%

a All metrics evaluated on 12 expert-curated queries using LLM-as-judge.

## 4 Conclusion and future work

Biomedical research increasingly requires synthesizing knowledge fragmented across thousands of publications to understand complex disease mechanisms and generate therapeutic hypotheses. In this work, we present eGoT, an end-to-end framework that addresses this challenge by constructing knowledge graphs directly from unstructured literature and enabling iterative multi-hop reasoning over the resulting graphs. Unlike prior approaches that require pre-existing curated knowledge bases or rely on text retrieval only, eGoT provides a complete pipeline from primary literature to knowledge graph construction and answer generation for queries. On standard benchmarks (HotpotQA, MultiHopRAG, Ultradomain), eGoT exceeds state-of-the-art performance on key metrics.

We demonstrate eGoT’s capabilities on two biomedical applications: question answering over 1046 small cell lung cancer publications, and cross-domain hypothesis generation linking lupus pathophysiology with UV radiation and climate factors. In the latter case, eGoT successfully traced mechanistic chains spanning up to 6 reasoning hops across traditionally siloed domains. This demonstrates the integrative reasoning required to understand environment-disease interactions that would be difficult to achieve with single-pass retrieval methods. Importantly, due to its architecture leveraging retrieval and knowledge graphs, every claim in eGoT responses is traced to specific papers, enabling clear provenance tracking and expert validation.

These capabilities are broadly applicable to other biomedical challenges requiring integration of molecular, clinical, and environmental knowledge, such as disease-environment interactions in cancer epidemiology or nutrition-microbiome-metabolic disease relationships. We anticipate eGoT will be particularly valuable for research areas requiring integration of heterogeneous knowledge sources, such as pharmacogenomics, where genetic variants must be connected to drug responses through molecular pathways, or systems biology approaches to complex diseases where environmental, immunological, and genetic factors interact. eGoT’s ability to construct domain-specific knowledge graphs from primary literature and reason across them, therefore, has the potential to accelerate hypothesis generation and literature-grounded discovery in biomedicine.

## Author contributions

Nihar Sanda (Conceptualization, Methodology, Software, Validation, Formal analysis, Investigation, Visualization, Writing—original draft, Writing—review & editing), Benjamin M. Gyori (Conceptualization, Methodology, Supervision, Writing—original draft, Writing—review & editing), Vito Quaranta (Conceptualization, Methodology, Validation), Auroop Ganguly (Conceptualization, Supervision), and Ayan Paul (Conceptualization, Methodology, Supervision, Project administration, Funding acquisition, Writing—original draft, Writing—review & editing)

## Supplementary Material

btag216_Supplementary_Data
