# Supplementary material for: eGoT: enhanced graph-of-thoughts for multi-hop knowledge retrieval and hypothesis generation in biomedicine
Source: Bioinformatics. 2026 Jul 7;42(Suppl 1):btag216. doi: 10.1093/bioinformatics/btag216 (PMC13341121; doi:10.1093/bioinformatics/btag216)
Supplement: btag216_Supplementary_Data [file btag216_supplementary_data.pdf]

Dependence of eGoT on LLMs

Graph Construction: In eGoT, graph construction is carried out using the *LLaMA-4 Scout* model, a 109B-parameter general-purpose, instruction-tuned language model. This process is applied to both the QFS and MultiHopQA tasks. For the embedding generation, we used the sentence-transformers/all-MiniLM-L6-v2 model from HuggingFace, which has a dimension size of 384.

Graph Retrieval: For the QFS tasks reported in Table 1, we employ the *DeepSeek-V3* model, which has 671B parameters. Following the setup in Zhang et al. (2024c), all baseline methods also generate answers using the DeepSeek-V3 model for fair comparison. For the QFS experiments discussed in Section C, we instead utilize the *GPT-4o* model, comparing its outputs on our dataset against those produced by DeepSeek-V3 in prior methods. In contrast, for the MultiHopQA tasks, answer retrieval is consistently performed using the *GPT-4o* model.

Computational costs for eGoT and the KG depend on the choice of LLM and can be computed from the number of API calls and tokens necessary. Each eGoT query consumes up to 10,000 tokens depending on the retrieval hyperparameters. The KG construction costs depend on the number of documents ingested into the KG.

The runtime for KG construction is primarily driven by the processing time for each publication. Processing a single publication takes around 2 minutes in the presented use cases, however, through parallelization, the KG construction time was about 5 seconds per paper overall. Responding to queries using eGoT typically ranges from 1-2 minutes in our case studies, depending on the specifics.

API Costs: The DeepSeek-V3 and OpenAI GPT-4o models are accessed directly through their respective proprietary APIs, ensuring optimal performance and compatibility with their native serving infrastructures. In contrast, the LLaMA-4-Scout model is hosted locally using the vLLM inference engine, which provides efficient parallelized serving with an observed throughput of approximately 80 tokens per second. The overall computational cost and latency are primarily determined by the number of API calls made during inference. This number, in turn, depends on two key parameters of the reasoning algorithm: the maximum depth and the top-k thoughts used during the search process. For instance, when the maximum depth is set to 2, and the top-k parameter is set to 2, the algorithm requires a total of 7 large language model (LLM) calls to complete the reasoning trajectory. However, this can be optimized by combining the thought generation and evaluation steps into a single prompt; the total number of LLM invocations can be reduced to 5, thereby decreasing both cost and latency without significantly compromising performance.

Limitations of knowledge graph extraction and entity standardization

The entity standardization pipeline we used reduces surface-form fragmentation and improves graph connectivity, but we acknowledge it may over-merge near-synonymous but distinct entities (e.g., "lupus nephritis" vs. "lupus") or under-merge entities with low lexical overlap despite semantic equivalence. However, the downstream impact of standardization errors on retrieval quality is not significant, but we plan to address this issue by integrating biomedical ontologies as canonical grounding in future work.

For graph construction, the quality is inherently bounded by the extraction LLM's accuracy. Errors like missed relations, hallucinated predicates, and incorrect entity boundaries may propagate into the KG and can affect downstream retrieval. However, we mitigate this to a certain extent through overlapping chunking (shared context windows between adjacent chunks), transitive inference rules (Eq. 4), and lexical relationship augmentation (R_{lex}).

Handling contradictory evidence is still an unaddressed challenge in this domain as resolution of contradictions cannot be done based on a frequency-based metric or any heuristic, because evolving knowledge can be confounded with incorrect statements in the absence of a human judge. We are working on metrics for the strength of evidence and hope that we can address this in future work.

We rely on LLM-as-judge for QFS evaluation following prior SOTA work (GraphRAG, LightRAG, HiRAG), which enables scalable pairwise comparison between answers but introduces model-specific biases in quality judgments. For the SCLC case study, questions were curated with domain expert input, but scoring remained automated. We acknowledge that human evaluation from biomedical experts assessing factual accuracy, clinical relevance, and reasoning coherence would strengthen the validity of our results. The win-rate framework partially mitigates positional bias by alternating answer order across comparisons. Human evaluation of a representative subset, alongside calibration of LLM judge reliability against expert ratings, is a concrete next step we plan to include in the journal version.

Ultradomain benchmarking with GPT-4o model

We also perform the benchmarking for the Ultradomain dataset using the GPT-4o model. The results in Table 1 show that the system is not reliant on the model. It performs arguably good with various powerful models as well.

Dataset Statistics

Table 2 presents comprehensive statistics of the datasets used in our experimental evaluation. We report the total number of tokens, which are computed using Byte-Pair Encoding tokenization. We also report the number of source passages and the number of question-answer pairs for each dataset. The datasets span diverse domains, including Agriculture (Agri), Legal, and Mixed-domain corpora, as well as established multi-hop reasoning benchmarks HotpotQA and MultiHopRAG.

Ground truth free evaluation metrics

This section provides detailed descriptions of the evaluation metrics we use to assess the performance of the SCLC Knowledge Graphs on eGoT

Faithfulness

Faithfulness measures the factual consistency between a generated response and its retrieved context. This metric evaluates whether all claims made in the response can be substantiated by the retrieved context. A higher faithfulness score indicates greater consistency between the response and the supporting context.

Table 1 *Comparative evaluation of eGoT against HiRAG, LightRAG, NaiveRAG, GraphRAG, KAG, and No-GoT baseline across Legal, Agriculture, and Mixed domains. We report percentage win rates of eGoT versus each competing method in pairwise comparisons on Comprehensiveness (Comp.), Empowerment (Emp.), Diversity (Div.), and Overall performance metrics. The graph construction phase was performed using LLaMA-4 Scout, while answer retrieval and generation were conducted using GPT-4o. Higher percentages indicate superior performance of eGoT over the baseline methods.*

	**Legal**		**Agriculture**		**Mix**	
	HiRAG	eGoT	HiRAG	eGoT	HiRAG	eGoT
Comp.	8.00	**92.00**	34.50	**65.50**	42.00	**58.00**
Emp.	3.00	**97.00**	6.00	**94.00**	16.00	**84.00**
Div.	17.50	**82.50**	46.50	**53.50**	30.00	**70.00**
Overall	5.50	**94.50**	25.00	**75.00**	30.00	**70.00**
	LightRAG	eGoT	LightRAG	eGoT	LightRAG	eGoT
Comp.	18.50	**81.50**	27.00	**73.00**	28.28	**71.72**
Emp.	3.00	**97.00**	3.50	**96.50**	18.69	**81.31**
Div.	38.00	**62.00**	30.50	**69.50**	21.72	**78.28**
Overall	12.50	**87.50**	18.00	**82.00**	20.71	**79.29**
	NaiveRAG	eGoT	NaiveRAG	eGoT	NaiveRAG	eGoT
Comp.	32.00	**68.00**	25.00	**75.00**	16.16	**83.84**
Emp.	4.50	**95.50**	4.00	**96.00**	9.09	**90.91**
Div.	17.50	**82.50**	29.50	**70.50**	9.60	**90.40**
Overall	14.00	**86.00**	15.00	**85.00**	9.60	**90.40**
	GraphRAG	eGoT	GraphRAG	eGoT	GraphRAG	eGoT
Comp.	48.00	**52.00**	35.00	**65.00**	33.33	**66.67**
Emp.	7.50	**92.50**	2.50	**97.50**	12.12	**87.88**
Div.	49.00	**51.00**	47.50	**52.50**	22.73	**77.27**
Overall	34.00	**66.00**	21.00	**79.00**	21.21	**78.79**
	KAG	eGoT	KAG	eGoT	KAG	eGoT
Comp.	2.50	**97.50**	0.50	**99.50**	4.55	**95.45**
Emp.	0.50	**99.50**	0.00	**100.00**	3.03	**96.97**
Div.	4.00	**96.00**	1.50	**98.50**	5.05	**94.95**
Overall	0.50	**99.50**	0.00	**100.00**	3.03	**96.97**

Table 2 *Comprehensive dataset statistics across evaluation domains. We report the total token count (using Byte-Pair Encoding), number of source passages, and question-answer pairs for Agriculture (Agri), Legal, Mixed-domain, HotpotQA, and MultiHopRAG datasets. In the Ultradomain domain-specific datasets (Agri, Legal) contain 100, while (Mix) contains 130 QA pairs each for controlled evaluation. The (HotpotQA, MultiHop) datasets include 1,000 QA pairs for a comprehensive assessment of multi-hop reasoning capabilities.*

**Metric**	**Agri**	**Legal**	**Mix**	**HotpotQA**	**MultiHop**
Tokens	1.9M	2M	602K	1.2M	991K
Passages	11	94	61	9,827	435
# QA Pairs	100	100	130	1,000	1,000

Implementation: The faithfulness score, $\mathcal{F}$, is computed using the following procedure:

1. Identify all claims present in the response
2. Verify each claim against the retrieved context to determine if it can be inferred from the provided information
3. Calculate the faithfulness score using:

$$\mathcal{F} = \frac{N_{\text{claims}}}{N_{\text{claims}}^{\text{total}}}, \tag{11}$$

where N_{claims} is the number of claims supported by retrieved context, and $N_{\text{claims}}^{\text{total}}$ is the Total number of claims in response.

LLM-Based context precision

Context Precision measures the proportion of relevant chunks within the retrieved contexts. It is calculated as the mean of Precision@K for each chunk in the context, where Precision@K represents the ratio of relevant chunks at rank k to the total number of chunks at rank k.

$$\text{Context Precision@}K = \frac{\sum_{k=1}^{K} (\text{Precision@}k \cdot v_k)}{N_K}, \tag{12}$$

where N_K is the Total number of relevant items in the top K results.

$$\text{Precision@}k = \frac{\text{true positives@}k}{\text{true positives@}k + \text{false positives@}k} \tag{13}$$

LLM-Based Implementation: This approach leverages an LLM to evaluate context relevance without requiring reference answers:

- Each retrieved chunk in `retrieved_contexts` is compared against the `response` using an LLM
- Each chunk is scored as either 0 (not useful) or 1 (useful) based on whether it contributed to generating the response
- The Precision@K formula is then applied to quantify the quality of the retrieved context

The evaluation follows a Context → Response verification paradigm, ensuring that the retrieved information directly supports the generated output.

Context Relevance

Context Relevance measures the pertinence of retrieved contexts to the user input. Higher scores indicate stronger alignment between the retrieved contexts and the user's query.

Implementation: The context relevance score, $\mathcal{C}_{\text{relevance}}$, is computed through the following process:

1. The LLM evaluates retrieved contexts using two distinct prompt templates (`template_relevance1` and `template_relevance2`)
2. Each template returns a relevance rating on a three-point scale:
   - **0**: Retrieved contexts are completely irrelevant to the user's query
   - **1**: Contexts are partially relevant
   - **2**: Contexts are completely relevant
3. Each rating is normalized to a $[0, 1]$ scale by dividing by 2
4. $\mathcal{C}_{\text{relevance}}$ is determined as follows:
   - If both ratings are valid: final score = average of normalized values
   - If only one rating is valid: final score = the valid normalized value

$$\mathcal{C}_{\text{relevance}} = \begin{cases} \frac{1}{2}\left(\frac{r_1}{2} + \frac{r_2}{2}\right) & \text{if both ratings valid} \\ \frac{r_{\text{valid}}}{2} & \text{if one rating valid} \end{cases} \tag{14}$$

where r_1 and r_2 are the ratings from template 1 and template 2, respectively.

Knowledge Graph Statistics

Table 3 and Table 4 present detailed statistics of the KGs constructed for each dataset using our eGoT framework. The graphs are hierarchically organized with three node types: Document nodes (representing the names of documents), Chunk nodes (representing document segments and their vector embeddings), and entity nodes extracted from the text. We report three types of relations: Document-to-Chunk relations (hierarchical connections), Chunk-to-Node relations (content associations between chunk nodes and entity nodes), and Node-to-Node relations (semantic relationships between 2 entity nodes). These statistics demonstrate the varying complexity and connectivity patterns across different domains, with the small cell lung cancer dataset exhibiting the highest graph density.

Table 3 *Node distribution statistics of KGs constructed using eGoT across evaluation datasets. We report the total node count and breakdown by node types (Document and Chunk nodes). Entity nodes can be derived as the difference between the total and the sum of Document and Chunk nodes. Graph construction was performed using LLaMA-4 Scout.*

Dataset	Total Nodes	Document Nodes	Chunk Nodes
Agriculture	26,335	11	1,498
Legal	24,466	94	3,676
Mix	11,408	61	501
HotpotQA	76,265	9,811	14,138
MultiHopRAG	5,417	466	467
SCLC	144,896	1,046	53,632
Lupus&UV	31,028	120	11812

Table 4 *Relation distribution statistics of KGs constructed using eGoT across evaluation datasets. Relations include Document-Chunk hierarchical links, Chunk-Node content associations, and Node-Node semantic relationships. The Node-Node relations dominate the graph structure across all datasets, indicating rich semantic connectivity. The SCLC dataset exhibits the highest complexity, with over 3 million relations, while MultiHopRAG shows the most compact structure suitable for controlled multi-hop reasoning evaluation.*

Dataset	Total Relations	Doc-Chunk Relations	Chunk-Node Relations	Node-Node Relations
Agriculture	1,110,423	1,498	60,817	1,019,571
Legal	544,556	3,676	72,620	433,212
Mix	85,214	501	13,549	60,678
HotpotQA	360,959	14,138	98,173	165,880
MultiHopRAG	12,678	467	3,144	5,908
SCLC	3,009,195	53,632	447,931	2,314,625
Lupus&UV	598,220	11,812	346,629	250,556

Dependence of eGoT on θ_a and θ_b

The eGoT retrieval pipeline is governed by two key thresholds: the answer relevance threshold θ_a, which controls the minimum relevance score required for a retrieved passage to contribute to answer generation, and the context relevance threshold θ_b, which filters the supporting context graph during multi-hop traversal. Together, these thresholds balance retrieval precision against coverage, and their interaction has a non-trivial effect on downstream generation quality.

To identify the optimal configuration, we performed an ablation study over four (θ_a, θ_b) pairs and evaluated each using three complementary RAG metrics: Faithfulness, LLM Context Precision, and NV Context Relevance. Faithfulness measures the degree to which generated answers are grounded in retrieved context; LLM Context Precision captures how much of the retrieved context is actually relevant to the query; and NV Context Relevance assesses the semantic alignment between retrieved passages and the original question.

As shown in Table 5, the configuration $\theta_a = 0.3$, $\theta_b = 0.9$ yields the best overall performance, achieving the highest Faithfulness (0.9428 ± 0.0944), LLM Context Precision (0.8571 ± 0.3586), and NV Context Relevance (0.9881 ± 0.0546). This suggests that relaxing the answer threshold while enforcing a stricter context filter is the most effective strategy: a lower θ_a allows the model to draw from a broader set of candidate answers, while a higher θ_b ensures the supporting graph remains tightly scoped to relevant evidence. Conversely, configurations with higher θ_a values (0.5 / 0.8, 0.5 / 0.9) show degraded LLM Context Precision, indicating that overly restrictive answer filtering prunes useful multi-hop paths before they can be fully traversed. We therefore adopt $\theta_a = 0.3$, $\theta_b = 0.9$ as the default eGoT configuration for all subsequent experiments.

Table 5 *Ablation study over eGoT retrieval thresholds (θ_a, θ_b). We report Faithfulness, LLM Context Precision, and NV Context Relevance scores across four configurations with depth and top-k as 2. The optimal configuration is highlighted in bold.*

Config (θ_a / θ_b)	Faithfulness	LLM context precision	NV context relevance
0.3 / 0.7	0.8709 ± 0.2529	0.5714 ± 0.5071	0.9643 ± 0.1195
**0.3 / 0.9**	$\mathbf{0.9428 \pm 0.0944}$	$\mathbf{0.8571 \pm 0.3586}$	$\mathbf{0.9881 \pm 0.0546}$
0.5 / 0.8	0.9007 ± 0.1507	0.8095 ± 0.4024	0.9881 ± 0.0546
0.5 / 0.9	0.8721 ± 0.2005	0.7143 ± 0.4629	0.9762 ± 0.0752

Qualitative Examples of Thought Generation.

We illustrate eGoT's iterative thought generation on two domain expert-curated SCLC questions below.

Q: *Elaborate on strategies that could inhibit phenotypic plasticity in SCLC.*

Depth 0, Thought 1: What are the key signaling pathways that contribute to phenotypic plasticity in SCLC, and how can they be targeted?
Depth 0, Thought 2: Can targeting cancer stem cells or specific cell subpopulations help inhibit phenotypic plasticity in SCLC?
Depth 1, Thought 1: What are the key differences in the molecular mechanisms underlying NE and non-NE subtypes of SCLC, and how can these differences be exploited to develop subtype-specific therapies?
Depth 1, Thought 2: Can inhibiting epithelial-mesenchymal transition (EMT) and other lineage plasticity mechanisms help reduce phenotypic plasticity in SCLC and improve therapeutic responsiveness?

Q: *What transcription factors drive an SCLC neuroendocrine phenotype?*

Depth 0, Thought 1: What is the role of ASCL1 in driving SCLC neuroendocrine differentiation?

Depth 0, Thought 2: Do transcription factors commonly associated with neuroendocrine tumors, such as NEUROD1 and INSM1, play a crucial role in the SCLC neuroendocrine phenotype?
Depth 1, Thought 1: What is the role of NEUROD1 in driving SCLC neuroendocrine differentiation, and how does it compare to ASCL1?
Depth 1, Thought 2: What is the relationship between ASCL1 and other neuroendocrine transcription factors, such as NEUROD1 and INSM1, in driving SCLC neuroendocrine differentiation?

Knowledge graph construction prompts

Entity extraction prompt

```
Your task: Read the text below (delimited by triple backticks) and identify all Subject-Predicate-Object (S-P-O) relationships in each sentence.
Then produce a single JSON array of objects, each representing one triple.
Follow these rules carefully:
- Entity Consistency: Use consistent names for entities throughout the document. For example, if "Systemic Lupus Erythematosus" is mentioned
as "sle", "lupus", and "rheumatic lupus" in different places, use a single consistent form (preferably the most complete one) in all triples. -
Atomic Terms: Identify distinct key terms (e.g., objects, diseases, locations, acronyms, people, conditions, concepts, feelings, environmental
concepts). Avoid merging multiple ideas into one term (they should be as "atomistic" as possible). - Unified References: Replace any pronouns
(e.g., "he," "she," "it," "they," etc.) with the actual referenced entity, if identifiable. - Pairwise Relationships: If multiple terms
co-occur in the same sentence (or a short paragraph that makes them contextually related), create one triple for each pair that has a meaningful
relationship. - CRITICAL INSTRUCTION: Predicates MUST be 1-3 words maximum. Never more than 3 words. Keep them extremely concise. - Ensure that
all possible relationships are identified in the text and are captured in an S-P-O relation. - Standardize terminology: If the same concept
appears with slight variations (e.g., "artificial intelligence" and "AI"), use the most common or canonical form consistently. - Make all the
text of S-P-O text lower-case, even Names of people and places. - If a person is mentioned by name, DO NOT create a relation to their location,
profession, and what they are known for (invented, wrote, started, title, etc.) if known.
Important Considerations: - Aim for precision in entity naming - use specific forms that distinguish between similar but different entities
- Maximize connectedness by using identical entity names for the same concepts throughout the document - Consider the entire context when
identifying entity references - ALL PREDICATES MUST BE 3 WORDS OR FEWER - this is a hard requirement
Output Requirements:
- Do not include any text or commentary outside of the JSON. - Return only the JSON array, with each triple as an object containing "subject",
"predicate", and "object". - Make sure the JSON is valid and properly formatted.
Example of the desired output structure:
[ { "subject": "Term A", "predicate": "relates to", // Notice: only 2 words "object": "Term B" }, { "subject": "Term C", "predicate": "uses", //
Notice: only 1 word "object": "Term D" } ]
Important: Only output the JSON array (with the S-P-O objects) and nothing else
Text to analyze (between triple backticks):
```

Community-based relationship inference prompt

```
I have a knowledge graph with two disconnected communities of entities.
Community 1 entities: entities1 Community 2 entities: entities2
Here are some existing relationships involving these entities: triples_text
Please infer 2-3 plausible relationships between entities from Community 1 and entities from Community 2. Return your answer as a JSON array of
triples in the following format:
[  "subject": "entity from community 1", "predicate": "inferred relationship", "object": "entity from community 2" , ... ]
Only include highly plausible relationships with clear predicates. IMPORTANT: The inferred relationships (predicates) MUST be no more than 3
words maximum. Preferably 1-2 words. Never more than 3. For predicates, use short phrases that clearly describe the relationship. IMPORTANT: Make
sure the subject and object are different entities - avoid self-references.
```

eGoT retrieval prompts

Thought generation prompt

```
You are an expert at reasoning and breaking down complex questions. Given the original question and the current state of gathered evidence,
generate a set of diverse and insightful next-step questions or hypotheses to explore. These "thoughts" should aim to uncover new information,
connect existing evidence, or validate hypotheses.
Original Question: question Current Evidence: evidence
Generate 2-4 distinct thoughts. Each thought should be a concise question or a statement to investigate. Return your response as a JSON object
with the following structure:  "thoughts": [ "thought 1", "thought 2", ... ]
```

Thought Evaluation Prompt

```
You are a critical thinker and evaluator. Given the original question and a set of generated "thoughts", evaluate the potential of each thought
to contribute to a comprehensive answer. Assign a score from 0.0 to 1.0 to each thought, where 1.0 is the most promising.
Original Question: question Generated Thoughts: thoughts
Return your response as a JSON object with the following structure:  "scores": [ "thought": "thought 1", "score": 0.9, "thought": "thought 2",
"score": 0.7, ... ]
```

Synthesis prompt

You are a precision synthesis expert who transforms validated evidence into comprehensive, actionable answers that excel in both depth and practical utility. Your responses must be accurate, thorough, and strictly grounded in the provided context.
Critical Constraint: You may ONLY use information from the provided context. Never use external knowledge or generate speculative answers.
WINNING RESPONSE FORMULA:
1. Specificity First - Start with specific examples, numbers, and data points from evidence - Include step-by-step procedures or methodologies when available - Name specific techniques, tools, people, or places mentioned - Provide exact measurements, percentages, or quantitative data - Use real-world applications and case studies from the evidence
2. Exhaustive Coverage - Cover the topic exhaustively using available evidence FIRST - Explain mechanisms, processes, and cause-effect relationships - Include historical context and development when present - Provide thorough practical implementation details - Only mention limitations after substantial content (80/20 rule)
3. Implementation Focus - Transform concepts into "how-to" guidance wherever possible - Include specific next steps readers can take - Explain practical applications alongside theoretical concepts - Provide concrete tools, resources, or methods from evidence - Frame information for immediate implementation
4. Integrated Analysis - Weave multiple viewpoints naturally throughout the response - Include varied evidence types without making gaps the focus - Present different angles while maintaining practical focus - Balance critical analysis with substantial content
5. Navigable Architecture - Lead with most actionable/concrete information - Build from specific examples to broader principles - Use clear headings for easy navigation - Progressive disclosure: essential → detailed → nuanced - Conclude with practical synthesis, not just gaps
SYNTHESIS APPROACH:
1. **First Pass - Concrete Foundation** - Extract ALL specific data, examples, numbers - Identify step-by-step processes or procedures - List concrete tools, methods, or applications
2. **Second Pass - Comprehensive Build** - Expand each concrete element with full context - Connect related evidence for complete picture - Add mechanisms and explanations
3. **Third Pass - Practical Integration** - Transform knowledge into actionable guidance - Highlight implementation pathways - Ensure every section has practical value
4. **Final Pass - Strategic Polish** - Verify 80% content / 20% limitations ratio - Ensure concrete examples appear early - Check that gaps enhance rather than dominate - Confirm actionable takeaways throughout
RESPONSE CHECKLIST: Before finalizing, ensure you have: - [ ] Specific numbers, names, or concrete examples in first paragraph - [ ] Step-by-step guidance or clear procedures - [ ] Exhaustive coverage of what IS known - [ ] Practical applications clearly explained - [ ] Multiple perspectives woven throughout (not separate) - [ ] Gaps/limitations as minor concluding notes only - [ ] Clear actionable takeaways for readers
Question: original_question
Context: <context> evidence_summary </context>
Remember: Win through DEPTH + DETAIL + ACTIONABILITY. Comprehensive answers with concrete specifics and practical guidance consistently outperform analytical frameworks alone. Your goal is to be the most useful possible resource while maintaining analytical rigor. "
